# Behçet’s disease and genetic interactions between HLA-B*51 and variants in genes of autoinflammatory syndromes

**DOI:** 10.1038/s41598-019-39113-5

**Published:** 2019-02-26

**Authors:** Sergio Burillo-Sanz, Marco-Antonio Montes-Cano, José-Raúl García-Lozano, Israel Olivas-Martínez, Norberto Ortego-Centeno, Francisco-José García-Hernández, Gerard Espinosa, Genaro Graña-Gil, Juan Sánchez-Bursón, María Rosa Juliá, Roser Solans, Ricardo Blanco, Ana-Celia Barnosi-Marín, Ricardo Gómez de la Torre, Patricia Fanlo, Mónica Rodríguez-Carballeira, Luis Rodríguez-Rodríguez, Teresa Camps, Santos Castañeda, Juan-Jose Alegre-Sancho, Javier Martín, María Francisca González-Escribano

**Affiliations:** 1 Department of Immunology, Hospital Universitario Virgen del Rocío (IBiS, CSIC, US), Sevilla, 41013 Spain; 2 Department of Internal Medicine, Hospital Clínico San Cecilio, Granada, 18003 Spain; 3 Department of Internal Medicine, Hospital Universitario Virgen del Rocío, Sevilla, 41003 Spain; 4 Department Autoimmune Diseases, Hospital Clinic Universitari, Barcelona, 08036 Spain; 5 Department of Rheumatology, Complejo Hospitalario Universitario, A Coruña, 15006 Spain; 6 Department of Rheumatology, Hospital Universitario de Valme, Sevilla, 41014 Spain; 7 Department of Immunology, Hospital Universitari Son Espases, Palma de Mallorca, Illes Balears, 07120 Spain; 8 Department of Internal Medicine, Autoimmune Systemic Diseases Unit, Hospital Vall d’Hebron, Universidad Autonoma de Barcelona, Barcelona, 08035 Spain; 9 Department of Rheumatology, Hospital Universitario Marqués de Valdecilla, Santander, 39008 Spain; 10 Department of Internal Medicine, Complejo Hospitalario Torrecárdenas, Almería, 04009 Spain; 11 Department of Internal Medicine, Hospital Universitario Central de Asturias, Asturias, 33011 Spain; 12 Department of Internal Medicine, Hospital Virgen del Camino, Pamplona, 31008 Spain; 13 Deparment of Internal Medicine, Hospital Universitari Mútua Terrassa, Terrassa, 08221 Spain; 14 Department of Rheumatology, Hospital Clínico San Carlos, Madrid, 28040 Spain; 15 Department of Internal Medicine, Hospital Regional Universitario, Málaga, 29010 Spain; 16 Department of Rheumatology, Hospital de la Princesa, IIS-Princesa, Madrid, 28006 Spain; 17 Department of Rheumatology, Hospital Universitario Doctor Peset, Valencia, 46017 Spain; 18 Instituto de Parasitología y Biomedicina “López-Neyra”, CSIC, PTS, Granada, 18016 Spain

## Abstract

Behçet’s disease (BD) is an immune-mediated systemic disorder with a well-established genetic base. In a previous study, using a next generation sequencing approach, we found many rare variants and some functional polymorphisms in genes related to autoinflammatory syndromes (AID): *CECR1*, *MEFV*, *MVK*, *NLRP3*, *NOD2*, *PSTPIP1* and *TNFRSF1A* in our BD cohort. Our strategy did not allow us to establish either number of patients with variants, proportion of individuals accumulating them or relationship with other genetic factors. With the goal to answer these questions, the individual samples were sequenced. Additionally, three functional polymorphisms: *NLRP3* p.Gln703Lys, *NOD2* p.Arg702Trp and p.Val955Ile were genotyped using TaqMan assays. A total of 98 patients (27.6%) carried at least one rare variant and 13 of them (3.7%) accumulated two or three. Functional regression model analysis suggests epistatic interaction between B51 and *MEFV* (P = 0.003). A suggestive protective association of the minor allele of *NOD2* p.Arg702Trp (P = 0.01) was found in both, B51 positive and negative individuals. Therefore, a high percentage of patients with BD have rare variants in AID genes. Our results suggest that the association of MEFV with BD could be modulated by the HLA molecules; whereas the protective effect of NOD2 p.Arg702Trp would be independent of HLA.

## Introduction

Behçet’s disease (BD) is a complex and systemic inflammatory syndrome; from a clinical point of view, it is a vasculitis that presents heterogeneous phenotypes. Genital and oral ulcers are the first and most common manifestations, although bilateral uveitis and cutaneous involvement are also present with a relatively high frequency, and there are occasional manifestations in other organs or systems, such as the nervous system^1–3^. Gender distribution is variable among countries, but unlike other immune-mediated diseases there is not a predominance of females among affected subjects^4^. BD is, in general, a rare disease although its prevalence is higher along the old “Silk Road”, especially in Turkey (up to 370 cases per 100000 population)^4^. The pathogenic mechanisms of BD remain unknown, although there are accumulated evidences of genetic predisposition and data suggesting that an aberrant immune response to certain infectious agents and some environmental factors may trigger the disease^5^. The first described genetic factor associated with the disease was HLA-B*51^6^, which is present in up to 60% of patients. More recently, genome wide association studies (GWAS) reinforce HLA-B*51 as the most important genetic factor in susceptibility to this disease and, in addition, they support the contribution of other genes related with the immune system, such as *IL23R*, *IL10*, *STAT4*, *CCR1-CCR3*, *KLRC4*, *ERAP1*, *TNFAIP3* and *FUT2*^7^.

There is a considerable clinical overlap of BD with autoinflammatory syndromes (AID) which are characterised by a misregulation of the inflammasome proteins that lead to an increased release of IL-1b. AID are genetically conditioned diseases, caused by aberrant proteins encoded by pathogenic variants of genes related with both the inflammasome itself (for example, NLRP3) as well as with other sensing proteins involved in pathogen- and damage-associated molecular patterns (PAMPs and DAMPs) or in the signalling pathways^8^. Heat shock proteins (HSP), are a group of intracellular proteins with scavenger roles found from bacteria to mammals. Human NF-M is homolog of bacterial HSP-65, and BD patients’ sera show crossreativity with these two proteins, suggesting that bacterial HSP could be recognised by TLRs and act as a trigger antigen for BD development^9,10^. Based on the above, it is plausible that variants in genes involved in AID could also be related to BD. In this sense, in a previous study, which included *CECR1*, *MEFV*, *MVK*, *NLRP3*, *NOD2*, *PSTPIP1* and *TNFRSF1A*, we reported that many rare variants in these AID related genes are found in BD patients^11^.

Gene-gene interactions are assumed to be a common factor in the genetic components of human diseases^12^, therefore, it is interesting to establish possible relationships among the different factors involved in the susceptibility to diseases. For instance, interaction between HLA and other genes associated with diseases has been demonstrated in BD and other immuno-mediated syndromes related to HLA class I, such as ankylosing spondylitis and psoriasis^7,13,14^. Our previous study with AID related genes was performed using next generation sequencing (NGS) with a pooled strategy which did not allow us to assign each variant to an individual. The aim of the present study was to determine the number of patients carrying variants, the proportion of individuals that accumulate variants and the relationship with other genetic factors, such as HLA-B*51.

## Materials and Methods

### Subjects

The study population consisted of 355 patients diagnosed with BD according the 1990 International Study Group classification criteria for this disease^15,16^. All the patients had Spanish European ancestry^16^, 43.7% were male and 36% were B51 positive. The clinical features of this cohort have been previously published^11^. The study was approved by the ethical committees of all centers and hospitals involved (Hospital Universitario Virgen del Rocío, Hospital Clínico San Cecilio, Hospital Universitari Clínic, Complejo Hospitalario Universitario A Coruña, Hospital Universitario de Valme, Hospital Universitari Son Espases, Hospital Vall d’Hebron, Hospital Universitario Marqués de Valdecilla, Complejo Hospitalario Torrecárdenas, Hospital Universitario Central de Asturias, Hospital Virgen del Camino, Hospital Universitari Mútua Terrassa, Hospital Clínico San Carlos, Hospital Regional Universitario de Málaga, Hospital de la Princesa, Hospital Universitario Doctor Peset, Instituto de Parasitología y Biomedicina López-Neyra). The healthy control population in the rare variants’ analysis, consisted on the whole genome sequences of 107 individuals from the Iberian population of Spain (IBS population), included in the phase 3 of the 1000 Genomes Project; whereas in polymorphisms analysis 363 in-house healthy controls were used.

### AID Related Genes Genotyping

In a previously published study with the same cohort, 54 rare variants (minor allele frequency, MAF < 0.05 in IBS population) were found in the 7 genes included in the study by using an NGS approach of pooled samples^11^. In the present study, in order to identify those patients carrying each variant, all the samples included in the pools which contained each rare variant were individually studied. 51 of the 54 rare variants were sequenced by Sanger and the remaining three (NLRP3 Val198Met: rs121908147, C__64676050_10, NOD2 Asn289Ser: rs5743271, C__26935007_10 and TNFRSF1A Arg121Gln: rs4149584, C__12029224_20) were TaqMan genotyped. In addition, to determine whether close variants (in the same exon) are in the same (*cis*) or in different (*trans*) chromosome, a Hexaprimer Amplification Refractory Mutation System PCR (H-ARMS PCR) was used^17^. The pairs of variants studied were: *MEFV* p.Leu110Pro and p.Glu148Gln, using the primers MEFV-H_ARMS_110_148-F1, R1, F2, R2, F3 and R3 and *NOD2* p.Arg311Trp and p.Arg703Cys with the primers NOD2-H_ARMS_311_703-F1, F2, R1 and R2 (Supplementary Table 1). In the case of *MEFV* H-ARMS, the following thermal-cycler conditions were used: 3 min at 95 °C followed by 35 cycles of: 20 s at 95 °C, 30 s at 65 °C and 45 s at 72 °C. In this case, an informative band of 145 bp is present only when both variants are in *trans* (Supplementary Fig. 1A). Regarding *NOD2*, a modified H-ARMS was used, since the 2 variants are more distant, a two-PCR reactions system was chosen (one with the pair of primers F1 and R1 and another with the pair F2 and R2). The thermal-cycler conditions were: 20 s at 95 °C, 30 s at 70 °C and 90 s at 72 °C. In this case, an informative band of 1228 bp in one of the PCR tubes is present only when both variants are in *trans* (Supplementary Fig. 1B).

Regarding the three functional polymorphisms found in the previous study, NLRP3 p.Gln703Lys, NOD2 p.Arg702Trp and p.Val955Ile were individually genotyped in all the samples included in those pools in which the variants had been detected. The following TaqMan assays were used: NLRP3 p.Gln703Lys (rs35829419, C__25648615_10), NOD2 p.Arg702Trp (rs2066844, C__11717468_20) and p.Val955Ile (rs5743291, C__25651076_10). Real-Time PCR assays were performed using TaqPath™ ProAmp™ Master Mix (Thermo Fisher Scientific, Waltham, MA), in a 7500 Fast Real-Time PCR System, according to manufacturer’s recommendations. No statistically significant differences in the allelic frequencies of the polymorphisms were observed between NGS and TaqMan assay results. The remaining 4 polymorphisms (MAF > 0.05 in IBS population) found in our previous study were excluded from the present study because they do not have a functional relevance.

Regarding the controls, genotypes of rare variants were retrieved from the IBS population of the 1000 Genomes Project database and they were imported to PLINK software for genotype counting and association testing. In addition, in the cases of the functional polymorphisms NLRP3 p.Gln703Lys, NOD2 p.Arg702Trp and p.Val955Ile, 363 in-house individuals were genotyped using the same TaqMan assays as in patients and used as local ethnically matched controls.

### HLA-B Low Resolution Genotyping

The locus HLA-B was genotyped in the 343 BD patients and the 363 local controls by using a standard low resolution (first set of two digits) PCR-SSOP Luminex method (LABType SSO One Lambda Inc., Canoga Park, CA). The remaining 12 patients could not be genotyped in HLA because of lack of material or its poor quality. Regarding the IBS controls, HLA-B genotype of IBS population of the 1000 genomes project was called at the same level of resolution as the BD patients using a pipeline optimised for OptiType software^18^. Briefly, chr6:29910247-31325022 hg19 region was retrieved from the mapped exome BAM files and converted to FASTQ with bamtools; the readings were aligned to all the available HLA allele sequences of IMGT/HLA with razerS3 and these alignments were analysed by OptiType software for HLA class I type calling. Data were visually inspected for proper coverage of HLA class I exons. The allelic frequency of HLA-B*51 by this method in IBS population (N = 107) was 0.09, which is not significantly different from that obtained in the general Spanish population with a standard HLA typing method (0.07)^19^.

### Statistical analysis

To analyse the relationship between HLA-B*51 and rare variants in the AID genes, BD patients were divided into two groups: B51 positive (assigned as affected phenotype) and B51 negative (assigned as non-affected phenotype) and the regions studied were those defined by the field refGene.name2. SKAT test implemented by Variant Tools software^20,21^ was used to analyse the distribution of rare variants in each of the genes included in the study in both patient groups. Additionally, to detect interactions between rare variants of each pair of AID genes as well as between each AID genes and HLA-B*51, a recently developed functional regression model was used with the R package FRGEpistasis^22^. This method has been used for quantitative traits^23^, but it is also suitable for the binary ones. Briefly, the cases/controls were encoded as 1/0, the AID genes variants were codified as 0 (wild-type) or 1 (variant) and also the B51 status (1 positive, 0 negative). P-values were adjusted (P_adj_) according to Bonferroni’s (considering 8 tests: 7 AID genes and B51), therefore P-values below 0.0063 were considered statistically significant. Type I error was estimated using a simulation with data retrieved from the 1000 genomes database (Supplementary Table 2). In addition, FRG analysis was also done in our BD patients and IBS controls with randomly assigned the phenotypes (affected and unaffected) (Supplementary Table 3). Q-Q plots of the p-values obtained in FRG with both, the real and the random data are displayed in Supplementary Fig. 2.

In regards to the disease association study of the 3 functional polymorphisms: NLRP3 p.Gln703Lys, NOD2 p.Arg702Trp and p.Val955Ile, each sample was encoded as 0, 1 and 2 according to the number of copies of the minor allele of each polymorphism carried by the individual. Allelic and genotypic distributions in dominant (for minor allele) and allelic models in BD patients and controls were compared with Χ2 in two by two contingency Tables with 1-degree freedom using PLINK software and P-values were corrected by Bonferroni’s adjustment considering 12 tests, P values < 0.004 were considered statistically significant and the Odds Ratios (ORs) were calculated with *fmsb* package for R. Finally, to analyse interaction between B51 and these functional polymorphisms, conditional logistic regression models were constructed by categorizing all the individuals according to the presence/absence of the B51 (dominant model) and the genotype of each polymorphism (dominant model for the minor allele) and they were analysed with Epi Info 2002. Additionally, the R package FRGEpistasis^22^ was also used to detect interaction of these polymorphisms with B51.

To quantify linkage disequilibrium (LD) when it was relevant, the R values were calculated in the patients and local controls by using PLINK, whereas in the EUR population, LD between variants was consulted in HaploReg v.4.1^24^. A descriptive circular layout figure, performed with circlize R package, was used to graphically represent the rare variants that are found together in same patient^25^.

### Ethical use of human participants statement

All participants signed a written informed consent to publish identified information prior to their enrolment in the study. All methods were performed after obtaining the signed informed consent from patients, and in accordance to our institution ethical committee: Hospital Universitario Virgen del Rocio’s ethical committee, and with the Helsinki Declaration of 1975, as revised in 1983.

## Results

**Figure 1:**
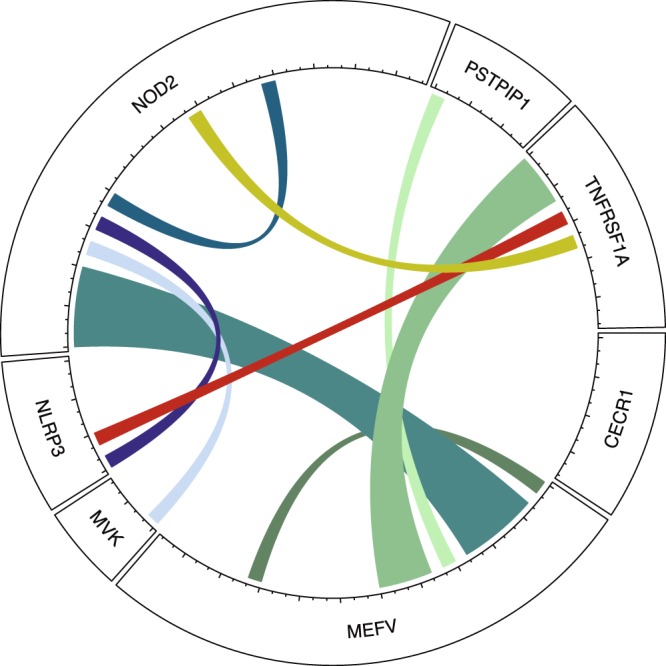
Descriptive circular layout showing coexistence of rare variants in patients. Length of sectors corresponds to number of variants; width of lines corresponds to the number of connections, each tick in inner circle axis represents one variant in one patient.

The individual genotyping of the samples revealed that 98 patients out of the total of 355 in our cohort (27.6%) carried at least one of the total of 54 rare variants found in the genes included in the study. From these 98 patients, 82 harboured only one rare variant, 13 had two and 3 had three, in the same or different genes. All the patients with one variant had a single copy, except two homozygous (one NOD2 p.Gly908Arg and the other TNFRSF1A p.Arg121Gln). In regard to the 13 patients carrying two variants, in 3 cases the variants were located in the same gene and in 10 cases they were in different genes. Regarding the 3 patients with 3 variants, 2 of them had the combined allele MEFV p.Arg408Gln/p.Pro369Ser and one variant in a different gene, whereas the remaining patient had each variant in a different gene. Figure 1 illustrates connections between variants and Table 1 displays the list of combinations found in our cohort. In order to know whether variants in the same gene were in *cis* or in *trans*, PCR-H-ARMS was performed in the patients with MEFV p.Glu148Gln/p.Leu110Pro and with NOD2 p.Arg703Cys/p.Arg311Trp. In the case of MEFV, the variants were in *cis* whereas in NOD2 they were in *trans* (Supplementary Fig. 1A,B). No additional study was made in the 3 individuals with the combination MEFV p.Arg408Gln/p.Pro369Ser since these variants have been reported as being in a strong LD in the European control population (r^2^ = 1), therefore *cis* configuration was assumed.

**Table 1:** List of rare variants found in combination in the seven AID genes studied in our cohort of BD patients.

Type of combination	Variant combination	Nr. Patients
Homozygous	NOD2 Gly908Arg^a^	1
	TNFRSF1A Arg121Gln	1
Variants in the same gene	MEFV Arg408Gln, Pro369Ser^b^	1
	MEFV Glu148Gln, Leu110Pro^c^	1
	NOD2 Arg703Cys, Arg311Trp^d^	1
Variants in different genes	MEFV Glu148Gln, PSTPIP1 Val122Ile	1
	MEFV Ile591Thr, NOD2 Gly908Arg	1
	MEFV Ile591Thr, TNFRSF1A Arg121Gln	1
	MEFV Met694Val, NOD2 Leu349Phe	2
	MEFV Lys695Arg, NOD2 Ala755Val	1
	MEFV Ala744Ser, TNFRSF1A Arg121Gln	1
	MVK Val377Ile, NOD2 Gly908Arg^a^	1
	NLRP3 Val198Met, TNFRSF1A Arg312Lys	1
	NLRP3 Arg488Lys, NOD2 Asn289Ser	1
	MEFV Glu148Gln, TNFRSF1A Arg121Gln, NOD2 Leu248Arg	1
	MEFV Arg408Gln, Pro369Ser, TNFRSF1A Arg121Gln^b^	1
	MEFV Arg408Gln, Pro369Ser, NOD2 Asn289Ser^b^	1

^a^Patient homozygous NOD2 Gly908Arg is the same as the one with the variant MVK Val377Ile.

^b^MEFV Arg408Gln and Pro369Ser are in a strong LD in the European control population (r^2^ = 1). A total of 3 patients have these two variants in our cohort.

^c^MEFV Leu110Pro / Glu148Gln LD has not been found in control population, but this combination has been reported as a complex allele in FMF and according the PCR-H-ARMS performed in this patient these variants are in *cis*.

^d^According to the results of the PCR-H-ARMS performed in this patient these variants are in *trans*.

The results of the SKAT test suggested non-significant differences in the distribution of rare variants between B51 positive and B51 negative patients (p > 0.05, Table 2). Nevertheless, functional regression model analysis, suggests epistatic interaction between HLA-B*51 and MEFV (p < 0.05, Table 2) in BD. No evidences of interaction between pairs of AID related genes were detected.

**Table 2:** Analysis of the distribution of rare variants in seven genes related to AID in B51 positive and negative BD patients using two different approaches.

Gene	SKAT P-value^a^ (N = 336)	FRG P-value^b^ (N = 443)
*CECR1*	0.77	0.51
*MEFV*	0.75	0.003
*MVK*	1.0	0.50
*NLRP3*	0.73	0.19
*NOD2*	0.99	0.77
*PSTPIP1*	0.82	0.40
*TNFRSF1A*	0.46	0.85

^a^B51 positive (N = 147) vs. B51 negative (N = 189) patients were compared using SKAT.

^b^BD patients (N = 336) vs. IBS 1000 Genomes controls (N = 107) were compared using FRG analysis for epistasis. Only the results of the epistasis analysis between B51 and the AID genes are displayed; although interactions between pairs of AID genes were also analysed, no interactions were detected (p-values > 0.05 in all the cases). Bonferroni’s adjusted significance level is p-value < 0.0063.

Table 3 displays the frequencies of the three functional polymorphisms in BD patients and healthy controls. A suggestive association was found in the case of NOD2 p.Arg702Trp (p = 0.011 in the allelic model and p = 0.014 in the dominant model: TT + CT vs. CC) with a protective effect of the minor allele. For the two remaining polymorphisms, no-significant association with BD was found (p > 0.05). No LD was detected between the two *NOD2* polymorphisms included (R < 0.1 in all cases). With respect to the relationship of these polymorphism with B51, conditional regression analysis suggests that the protective effect of NOD2 p.Arg702Trp is independent of the HLA-B51, because the OR is decreased in both, B51 positive and negative groups (Table 4). The functional regression model analysis does not suggest epistatic interaction between B51 and these polymorphisms (p > 0.05 in all the cases).

**Table 3:** Distribution of frequencies of NLRP3 and NOD2 functional polymorphisms in BD cases and healthy controls.

		Patients		Healthy Controls		P	OR (95% CI)
		BD N = 355	Local N = 363	IBS N = 107	Overall^a^		
NLRP3 Gln703Lys	A/A	0 (0%)	0 (0%)	3 (2.8%)	3 (0.6%)		
	C/A	32 (9.0%)	32 (8.8%)	10 (9.3%)	42 (8.9%)		
	C/C	322 (91.0%)	331 (91.2%)	94 (87.8%)	425 (90.4%)		
	A	32 (0.05)	32 (0.04)	16 (0.08)	48 (0.05)	0.58	0.88 (0.56–1.39)
	C	676 (0.95)	694 (0.96)	198 (0.92)	892 (0.95)		
NOD2 Val955Ile	A/A	8 (2.3%)	2 (0.6%)	4 (3.7%)	6 (1.3%)		
	A/G	51 (14.4%)	58 (16%)	20 (18.7%)	78 (16.6%)		
	G/G	296 (83.4%)	303 (83.4%)	83 (77.6%)	386 (82.1%)		
	A	67 (0.09)	62 (0.09)	28 (0.13)	90 (0.1)	0.92	0.98 (0.71–1.37)
	G	643 (0.91)	664 (0.91)	186 (0.87)	850 (0.9)		
NOD2 Arg702Trp	T/T	0 (0%)	1 (0.3%)	0 (0.0%)	1 (0.2%)		
	C/T	18 (5.0%)	33 (9.1%)	12 (11.2%)	45 (9.6%)		
	C/C	336 (95.0%)	329 (90.6%)	95 (88.8%)	424		
					(90.2%)		
	T	18 (0.03)	35 (0.05)	12 (0.06)	47 (0.05)	0.011	0.496 (0.29–0.86)
	C	690 (0.97)	691 (0.95)	202 (0.94)	893 (0.95)		

^a^No differences in allelic frequencies between local and IBS 1000 genomes controls were found, therefore, overall control data were used to calculate p-values and ORs. Bonferroni’s adjusted significance level considered significant is P-values < 0.004.

**Table 4:** Conditional logistic regression model of HLA-B51 and NOD2 p.Arg702Trp.

HLA B51	NOD2 Arg702Trp	Patients	Controls	P	OR (95 % CI)
+	+	10 (3.0)	5 (1.4)	0.04	3.11 (1.04–9.23)
+	−	136 (40.6)	46 (12.7)	<10^−5^	4.59 (3.13–6.73)
−	+	8 (2.4)	27 (7.5)	0.06	0.46 (0.20–1.03)
−	−	181 (54.0)	284 (78.4)	<10^−5^	1.0

## Discussion

In the present study, a substantial percentage (about 30%) of the BD patients was found to have some rare variant in at least one out the seven AID related genes that were included. The proteins encoded by these AID genes are related with the IL-1b release, mainly through sensing bacterial or viral components and activating the NLRP3 inflammasome and, therefore, it would be reasonable to expect some degree of interaction among them. In this way, a relatively high ratio of our BD patients carried variants in different AID genes, suggesting that interplay between variants might exist. Hypothetically, these variants in AID genes could modify the association of B51 with BD or vice versa. It is necessary to note that, genetic interaction is difficult to detect because multilocus genotype combinations for gene-gene interaction increase exponentially and require a larger sample size as well as more computation burden. In addition, most of the methods developed to detect gene-gene interactions have been designed for common variants and it is difficult to apply them to data with rare variants because the low frequency of these variants leads to a lack of statistical power^26,27^. In spite of the use of a method designed for rare variants, no-interactions between AID genes were detected, although this result could be attributable to the low sample size, since only 13 patients accumulate variants in different genes. In addition, it is necessary to take into account that, from a biological point of view, gene-gene interaction is the phenomenon in which the effects of one gene on a trait (for example a disease) are modified by another gene or several other genes; whereas statistical epistasis is described as a deviation from additivity in a linear statistical model^28^. In order to assess interaction between rare variants of AID genes and HLA-B*51, two different approaches were used; in the first, patients B51 positive and negative were compared with SKAT whereas in the second, patients and controls were compared using FRG. The only discrepancy observed between both tests was in the case of *MEFV* in which, both tests have apparently contradictory results. According to SKAT, no differences in the distribution of rare variants were detected between B51 positive and negative individuals whereas our results with FRG suggest interaction between HLA-B*51 and *MEFV*. The estimated type I error in our simulation is even lower for FRG than for SKAT therefore, discrepancy could be based in the characteristics of each test, SKAT was designed to study association of rare variants in complex diseases by grouping variants and checking the association of each entire gene, whereas FRG was specifically designed to study gene-gene interactions with rare variants. Contradictory results in the association of *MEFV* with BD have been reported^29–32^, in fact, in our previous study in AID genes, *MEFV* was found associated only with SKAT but not with the rest of the statistical tests used^11^. To our acknowledgement, no previous work on interaction between HLA and *MEFV* has been published for BD. Our results must be interpreted with caution, nevertheless, there is some evidence suggesting that involvement of *MEFV* in autoimmune diseases could be associated with HLA molecules^33–35^ and conversely, differential effects of HLA class I molecules in Japanese patients with familial Mediterranean fever (FMF), even in those with high-penetrance MEFV mutations, have been identified^36^. MEFV protein interacts with cellular components of inflammasome whose activity is augmented by the pathogenic mutations^37^. Therefore, it is reasonable to consider that FMF-associated *MEFV* mutations enhance the cellular responses in the inflammatory diseases which are also associated with certain HLA polymorphisms. In other words, mutations and polymorphisms in the *MEFV* gene are associated with subclinical inflammation therefore, it is possible that the presence of *MEFV* mutations increase the baseline level of inflammation, and the presence of a certain HLA molecule (such as HLA-B*51 in BD) modulates the disease. Although the same type of interaction could be hypothesized for any of the other AID genes studied, no more interactions were detected.

Regarding the polymorphisms in *NLRP3* and *NOD2*, only a protective association of NOD2 p.Arg702Trp was found. The loss of function variants of NOD2 have been reported as possibly protective in BD^30,38^. For this reason, in our previous study, the frequencies of NOD2 p.Asn289Ser, p.Arg702Trp and p.Gly908Arg in our patient cohort were compared with those of the 1000 genomes IBS control population^11^. Although no significant differences were found, a trend was detected for p.Arg702Trp (p = 0.09) that in the present study reaches statistical significance by increasing the number of controls by including local ethnically matched individuals. Therefore, the present study suggests a protective effect of this *NOD2* variant at least in Caucasian patients. This effect seems to be independent of HLA-B*51.

In conclusion, a high percentage of patients with BD have one or several variants in AID genes. Additionally, although our data require replication, they suggest that the association of *MEFV* with BD could be modulated by the HLA molecules; whereas the protective effect of the loss of function of NOD2 p.Arg702Trp polymorphism would be independent of HLA.

## Supplementary information

Supplementary Material for Behçet’s disease and genetic interactions between HLA-B*51 and variants in genes of autoinflammatory syndromes
