# Supplementary material for: Behçet’s disease and genetic interactions between HLA-B*51 and variants in genes of autoinflammatory syndromes
Source: Sci Rep. 2019 Feb 26;9:2777. doi: 10.1038/s41598-019-39113-5 (PMC6391494; doi:10.1038/s41598-019-39113-5)

Behçet's disease and genetic interactions between HLA-B*51 and variants in genes of autoinflammatory syndromes

Sergio Burillo-Sanz[1], Marco-Antonio Montes-Cano[1], José-Raúl García-Lozano[1], Israel Olivas-Martínez[1], Norberto Ortego-Centeno[2], Francisco-José García-Hernández[3], Gerard Espinosa[4], Genaro Graña-Gil[5], Juan Sánchez-Bursón[6], María Rosa Juliá[7], Roser Solans[8], Ricardo Blanco[9], Ana-Celia Barnosi-Marín[10], Ricardo Gómez de la Torre[11], Patricia Fanlo[12], Mónica Rodríguez-Carballeira[13], Luis Rodríguez-Rodríguez[14], Teresa Camps[15], Santos Castañeda[16], Juan-Jose Alegre-Sancho[17], Javier Martín[18], María Francisca González-Escribano[1]*.

[1]Department of Immunology, Hospital Universitario Virgen del Rocío (IBiS, CSIC, US), Sevilla 41013, Spain. [2]Department of Internal Medicine, Hospital Clínico San Cecilio, Granada 18003, Spain. [3]Department of Internal Medicine, Hospital Universitario Virgen del Rocío, Sevilla 41003, Spain. [4]Department Autoimmune Diseases, Hospital Universitari Clínic, Barcelona 08036, Spain. [5]Department of Rheumatology, Complejo Hospitalario Universitario A Coruña, A Coruña 15006, Spain

[6]Department of Rheumatology, Hospital Universitario de Valme, Sevilla 41014, Spain

[7]Department of Immunology, Hospital Universitari Son Espases, Palma de Mallorca 07120, Spain. [8]Department of Internal Medicine, Autoimmune Systemic Diseases Unit, Hospital Vall d'Hebron, Universidad Autonoma de Barcelona, Barcelona 08035, Spain.

[9]Department of Rheumatology, Hospital Universitario Marqués de Valdecilla, Santander 39008, Spain. [10]Department of Internal Medicine, Complejo Hospitalario Torrecárdenas, Almería 04009, Spain. [11]Department of Internal Medicine, Hospital Universitario Central de Asturias, Asturias 33011, Spain. [12]Department of Internal Medicine, Hospital Virgen del Camino, Pamplona 31008,

Spain. [13]Deparment of Internal Medicine, Hospital Universitari Mútua Terrassa, Terrassa 08221, Spain. [14]Department of Rheumatology, Hospital Clínico San Carlos, Madrid 28040, Spain. [15]Department of Internal Medicine, Hospital Regional Universitario de Málaga, Málaga 29010, Spain. [16] Department of Rheumatology, Hospital de la Princesa, IIS-Princesa, Madrid 28006, Spain. [17]Department of Rheumatology, Hospital Universitario Doctor Peset, Valencia 46017, Spain. [18]Instituto de Parasitología y Biomedicina "López-Neyra", CSIC, PTS Granada, Granada 18016, Spain.

Corresponding autor: María Francisca González-Escribano, Servicio de Inmunología. HU Virgen del Rocío. 41013 Sevilla. Spain. mariaf.gonzalez.sspa@juntadeandalucia.es. Tel +34955013228 Fax +34955013221.

Supplementary Table 1. List of primers used in H-ARMS PCR analysis

Assay	Primer Name	5' – 3' sequence
**MEFV Leu110Pro and Glu148Gln H-ARMS**	F1	CGCCCGGCCCGTTGTTTTCCTCAATTTC
	R1	GCAGGGCCGGGCTCCGGGTCCGAGGCTT
	F2	CCCTGGGGGAGAACAAGCCCAGGAGACC
	R2	CCCCTCGGGGTGGTCTGGAGTCTGCA
	F3	CTGCCAGCCTGCGGTGCAGCCAGCGCC
	R3	GGGCTTCCTCGACAGCCCCCTCCCGGCGTC
**NOD2 Arg311Trp and Arg703Cys H-ARMS**	F1	TGGAGGAGCTCTTCAGCACCCCTGGCCA
	R1	CAGGCCACGTGCAGCCTTCCGAGCCAGCC
	F2	CAGTGGCAAGAGCACGCTCCTGCCGC
	R2	ACCCGGTGCAGCTGGCGGGATGGAGGGA

A)

MEFV

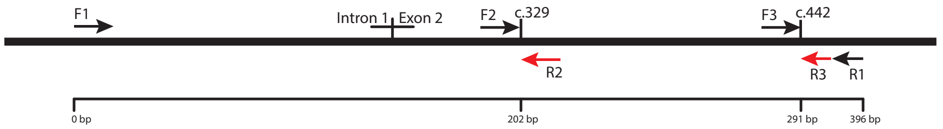

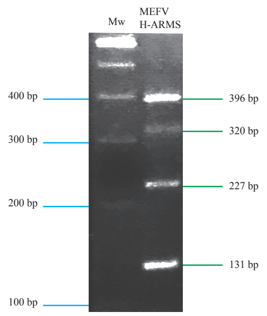

MEFV H-ARMS	
cis **: ab + AB haplotypes**	***trans*: Ab + aB haplotypes**
396	396
321	320
227	227
131	131
No band	145*

B)

NOD2

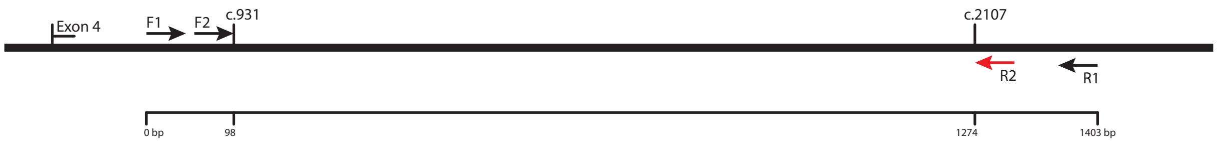

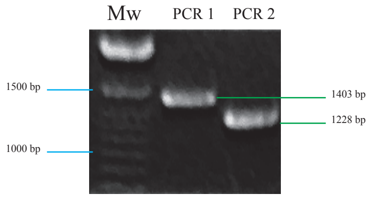

	NOD2 H-ARMS	
	***cis*: ab + AB haplotypes**	***trans*: Ab + aB haplotypes**
PCR 1 (F1 and R1 primers)	1403	1403
PCR 2 (F2 and R3 primers)	No band	1228*

C)

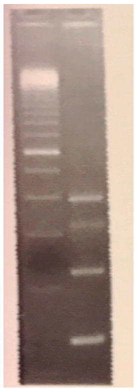

D)

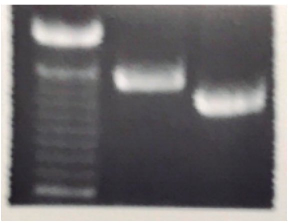

Supplementary Figure 1. Schematic representation of the H-ARMS PCR carried out in order to know the *cis*/*trans* configuration of two pairs of rare variants in *MEFV* and *NOD2* found in two BD patients. A) *MEFV* Leu110Pro and Glu148Gln analysis, uncropped electrophoresis gel showing all available bands. B) *NOD2* Arg311Trp and Arg703Cys analysis, electrophoresis gel cropped at 800 bp band of the molecular weight marker. C) Untouched A gel electrophoresis. D) Untouched B gel electrophoresis

Supplementary Table 2. Type I error estimations.

		Unadjusted significance level (α)	
		**0.05**	**0.01**
**Parameter**	**SKAT P-value**	0.088	0.013
	**FRG P-value**	0.014	<0.01

For estimation of type I error, a simulation was used. A total of 2596 rare variants of the genomic region hg19 chr22:17000000-31400001 were retrieved from 107 IBS and 107 TSI individuals from 1000 genomes database and HLA-B51 status was determined with OptiType software. Phenotype (case/control) was randomly assigned. Simulation data were analysed as it was done for samples using SKAT and FRG.

Supplementary Table 3. FRG control with random phenotypes.

Gene	FRG P-value (N=443)
CECR1	0.62
MEFV	0.70
NOD2	0.79
MVK	0.27
TNFRSF1A	0.84
NLRP3	0.14
PSTPIP1	0.68

BD patients and IBS 1000 genome controls were randomly assigned as affected or unaffected. Phenotype groups were compared using FRG analysis for epistasis of the studied genes with HLA-B51.

Supplementary Figure 2. QQ-Plots of FRG P-values. **A.** QQ-Plot constructed with FRG P-values from Table 2. **B.** QQ-Plot constructed with FRG P-values from Supplementary Table 3.

A.

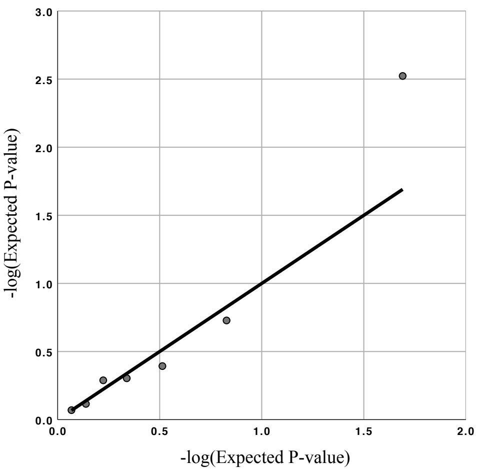

B.

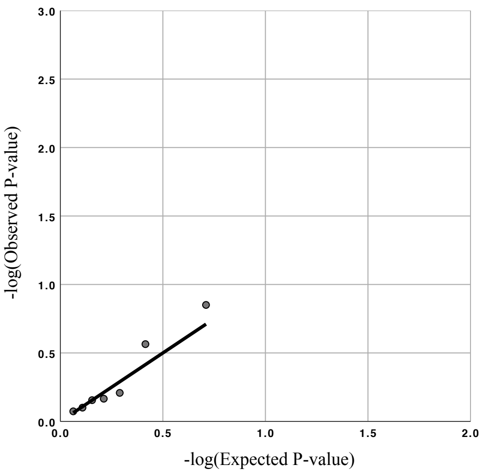

Supplement: Supplementary file 1 — Supplementary Material for Behçet’s disease and genetic interactions between HLA-B*51 and variants in genes of autoinflammatory syndromes [file 41598_2019_39113_MOESM1_ESM.pdf]
